# Novel lnc RNA regulated by HIF‐1 inhibits apoptotic cell death in the renal tubular epithelial cells under hypoxia

**DOI:** 10.14814/phy2.13203

**Published:** 2017-04-18

**Authors:** Imari Mimura, Yosuke Hirakawa, Yasuharu Kanki, Natsuki Kushida, Ryo Nakaki, Yutaka Suzuki, Tetsuhiro Tanaka, Hiroyuki Aburatani, Masaomi Nangaku

**Affiliations:** ^1^ Division of Nephrology and EndocrinologyGraduate School of MedicineThe University of TokyoTokyoJapan; ^2^ Isotope Science CenterThe University of Tokyo.TokyoJapan; ^3^ Division of GenomeScienceResearch Center for Advanced Science and TechnologyThe University of TokyoTokyoJapan; ^4^ Graduate School of Frontier SciencesThe University of TokyoTokyoJapan

**Keywords:** Apoptosis, HIF‐1, hypoxia, lncRNA, tubular cells

## Abstract

Chronic tubulointerstitial hypoxia plays an important role as the final common pathway to end‐stage renal disease. HIF‐1 (hypoxia‐inducible factor‐1) is a master transcriptional factor under hypoxia, regulating downstream target genes. Genome‐wide analysis of HIF‐1 binding sites using high‐throughput sequencers has clarified various kinds of downstream targets and made it possible to demonstrate the novel roles of HIF‐1. Our aim of this study is to identify novel HIF‐1 downstream epigenetic targets which may play important roles in the kidney. Immortalized tubular cell lines (HK2; human kidney‐2) and primary cultured cells (RPTEC; renal proximal tubular cell lines) were exposed to 1% hypoxia for 24–72 h. We performed RNA‐seq to clarify the expression of mRNA and long non‐coding RNA (lncRNA). We also examined ChIP‐seq to identify HIF‐1 binding sites under hypoxia. RNA‐seq identified 44 lncRNAs which are up‐regulated under hypoxic condition in both cells. ChIP‐seq analysis demonstrated that HIF‐1 also binds to the lncRNAs under hypoxia. The expression of novel lncRNA, DARS‐AS1 (aspartyl‐tRNA synthetase anti‐sense 1), is up‐regulated only under hypoxia and HIF‐1 binds to its promoter region, which includes two hypoxia‐responsive elements. Its expression is also up‐regulated with cobalt chloride exposure, while it is not under hypoxia when HIF‐1 is knocked down by siRNA. To clarify the biological roles of DARS‐AS1, we measured the activity of caspase 3/7 using anti‐sense oligo of DARS‐AS1. Knockdown of DARS‐AS1 deteriorated apoptotic cell death. In conclusion, we identified the novel lncRNAs regulated by HIF‐1 under hypoxia and clarified that DARS‐AS1 plays an important role in inhibiting apoptotic cell death in renal tubular cells.

## Introduction

HIF‐1 (hypoxia‐inducible factor ‐1) is a well‐known master transcriptional factor which binds to the regulatory regions of downstream target genes (Semenza et al. 1991, 1996; Semenza and Wang 1992; Wang and Semenza 1995; Wang et al. 1995; Semenza 2010). HIF *α*‐subunit makes a complex with HIF *β*‐subunit and binds to the hypoxia‐responsive elements (HRE) of the target genes (Wang and Semenza 1993; Jiang et al. 1996). Alpha‐subunits have three isoforms, HIF‐1*α*, HIF‐2*α*, and HIF‐3*α*. Degradation of *α*‐subunit is regulated by prolyl hydroxylase in an oxygen‐dependent manner. In hypoxic condition, *α*‐subunit accumulates in the cytosol because prolyl hydroxylase cannot work. Alpha‐subunit forms a dimer with a *β*‐subunit, shifts into the nucleus (Kaelin and Ratcliffe 2008), and regulates downstream target genes. In the kidney, our group and others have demonstrated that tubulointerstitial hypoxia is the final common pathway to end‐stage renal disease and that HIF‐1 plays important roles in the kidney (Manotham et al. 2004; Tanaka et al. 2004, 2005a,b, 2006; Nangaku 2006; Mimura and Nangaku 2010; Tanaka 2016).

Genome‐wide analysis using high‐throughput sequencers is useful to identify novel HIF‐1 downstream targets which play important roles to regulate gene expressions under hypoxia. We previously identified novel HIF‐1 downstream target genes under hypoxia using chromatin immunoprecipitation sequencing (ChIP‐seq) and microarray (Mimura et al. 2012; Shoji et al. 2013; Inoue et al. 2014; Kushida et al. 2016). HIF‐1*α* co‐operates with one of histone demethylases, lysine‐specific demethylase 3A (KDM3A), and regulates chromosome conformation to regulate downstream target gene, solute carrier family member 2A3 (*SLC2A3*) (Mimura et al. 2012).

High‐throughput genomic technologies also make it possible to detect novel transcripts on the genome (Mimura et al. 2014, 2014). The majority of the transcripts do not derive from annotated protein‐coding genes. Long non‐coding RNA (lncRNA) is defined as those >200 nucleotides in length. The characteristics of lncRNA are low levels of expression and high specificity of cell lines. In addition, lncRNAs interfere with tissue homeostasis and have roles in pathological processes (Lorenzen and Thum 2016).

There are some reports which examined the roles of lncRNAs under hypoxia. H19, which is one of the imprinted genes, was elevated under hypoxic stress by HIF‐1 and possessed oncogenic properties (Matouk et al. 1803). Another group showed that H19 lncRNA knockdown diminished HUVEC ability to form capillary structures on matrigel, suggesting a crucial role of H19 lncRNA in endothelial cells (Voellenkle et al. 2016). Moreover, genome‐wide analysis using RNA‐seq revealed that 122 lncRNAs including H19, MIR210HG, and metastasis‐associated lung adenocarcinoma transcript 1 (MALAT1) were induced in HUVECs under hypoxia and animal models of hindlimb ischemia (Voellenkle et al. 2016).

Yang et al. (2014) demonstrated that lincRNA‐p21, a hypoxia‐responsive lncRNA, is necessary for hypoxia‐enhanced glycolysis. Another lncRNA, MALAT1, was reported to enhance arsenite‐induced glycolysis through HIF‐1*α* stabilization (Luo et al. 1862). AK058003, another lncRNA induced by hypoxia, was reported to promote migration and invasion of gastric cancer cells both in vivo and in vitro (Wang et al. 2014). Role of lncRNAs in glycolysis, cell migration and invasion suggests that lncRNAs can be good therapeutic targets in cancer. However, a role of lncRNAs in the kidney remains unknown.

In this study, we examined hypoxia‐inducible lncRNAs by genome‐wide analysis and clarified roles of HIF‐1 downstream target lncRNAs under hypoxia in renal tubular cells.

## Materials and Methods

### Cell culture

HK2 (human kidney‐2: ATCC CRL‐2190) was purchased from ACTT. (Tokyo, Japan). HK2 cells were cultured in Dulbecco's modified Eagle's medium with F12 (Wako, Osaka, Japan) supplemented with 10% heat‐inactivated fetal bovine serum (FBS). Renal Proxymal Tubular Epithelial Cells (RPTECs: CC2553) (Lonza, Japan) were cultured in EVM supplemented with 0.5% FBS. Cells were grown in a humidified atmosphere with 5% CO_2_ at 37°C. The hypoxic condition (1% O_2_ for 72 h for HK2 and 48 h for RPTEC) was brought about by means of a hypoxic cultivation incubator (APM‐30D, ASTEC, Fukuoka, Japan).

### RNA isolation and Reverse Transcription PCR

Total RNA of cells was isolated using RNAiso Plus (Takara, Shiga, Japan) according to the manufacture's protocol. First‐strand cDNA was synthesized utilizing PrimeScript RT reagent Kit (Perfect Real Time) (Takara, Shiga, Japan).

### RT‐qPCR

The purified mRNA or ChIP samples were quantified by qPCR. qPCR was performed by KAPA SYBR FAST qPCR Kit (Nippon Genetics, Tokyo, Japan) on the CFX96 Touch (Bio‐Rad Hercules, CA). The expression of each gene was normalized by be‐ta actin. The sequences of primers used for qPCR are listed in Table 1.

**Table 1:** The list of primer sets

Name		
SLC2A1	Forward	CTTCACTGTCGTGTCGCTGT
	Reverse	CCAGGACCCACTTCAAAGAA
be‐ta actin	Forward	TCCCCCAACTTGAGATGTATGAAG
	Reverse	AACTGGTCTCAAGTCAGTGTACAGG
DARS‐AS1	Forward	AGCCAAGGACTGGTCTCTTTT
	Reverse	CTGTACTGGTGGGAAGAGCC
HIF1A	Forward	TGGCTGCATCTCGAGACTTT
	Reverse	GAAGACATCGCGGGGAC

### Knockdown of HIF1 by siRNA and DARS‐AS1 by anti‐sense oligo transfection

Cells were passaged in 6‐well plates and transfected with stealth RNA targeting human HIF1A and DARS‐AS1 (Thermo Fisher Scientific, Waltham, MA), or negative control nucleotide by using Lipofectamine^®^ RNAiMAX Reagent (Thermo Fisher Scientific, Waltham, MA) and Opti‐MEM^®^ (Thermo Fisher Scientific, Waltham, MA). Six hours after the transfection, the medium was exchanged, and hypoxic stimulation started after 48 h since siRNA or anti‐sense oligo transfection.

### Immunoprecipitation (ChIP)

Briefly, HK2 were cross linked for 10 min using 1% paraformaldehyde and sonicated into fragments. The samples were immunoprecipitated with 4 *μ*g of antibodies against HIF‐1*α* (NB100‐134, Novus Biologicals, Minneapolis, MN). We used Protein A sepharose beads (GE Healthcare, 17‐5138‐01) to immunoprecipitate samples. The details are described in the previous paper (Mimura et al. 2012; Kushida et al. 2016).

### RNA‐seq and ChIP‐seq sample analysis

We isolated mRNA as described above. RNA‐seq libraries were prepared and sequenced using the HiSeq platform (Illumina, San Diego, CA) according to the manufacturer's protocol. The reads per kilobase of exon per million mapped reads (RPKM) of each gene was calculated based on the length of the gene and the read counts mapped to the gene. ChIP‐seq samples were sequenced by Genome Analyzer II (Illumina, San Diego, CA). The sequences were aligned using human reference genome (UCSC hg19) using ELAND (Illumina, SanDiego, CA) (Freese et al. 2016). Details were described in our previous paper (Mimura et al. 2012; Kushida et al. 2016).

### Caspase 3/7 assay

We measured caspase‐3/7 activity using ‘The Caspase‐Glo 3/7 Assay kit’ (Promega, WI). The assay provides a luminogenic caspase‐3/7 substrate. Luminescence is proportional to the amount of caspase activity. We use multiwell‐plate in 96 wells. First, we knocked down the expression of *DARS‐AS1* using anti‐sense oligo. After we reseeded the HK2 cells at the amount of 1 × 10^5 per well in 96 well plate, we cultured cells under hypoxic condition (1%, 72 h). Then, we added the equal volume of caspase‐glo reagent, which is a mixture of caspase‐glo substrate and caspase‐glo buffer, as the medium per well. After we incubate 30 min, we measured luminescence using luminometer (PerkinElmer, Inc. MA).

### Gene Ontology

Official gene symbols of selected genes were subjected to The Database for Annotation, Visualization and Integrated Discovery (DAVID) v6.7 to obtain biological processes related to the genes. Human default background was used.

### Data access

Data were analyzed according to the minimum information about a microarray experiment (MIAME) rule. The data indicated in this publication are accessible through National Center for Biotechnology Information; Gene Expression Omnibus (DRA003786 and DRA003787) for RNA‐seq.

### Statistical analysis

Data are shown as mean ± S.D. *P*‐values were calculated using two‐tailed unpaired Student's t test. *P* < 0.05 was considered significant.

## Results

### Genome‐wide analysis of RNA‐seq in tubular cells identified hypoxia‐inducible genes and lncRNAs under hypoxia

**Figure 1:**
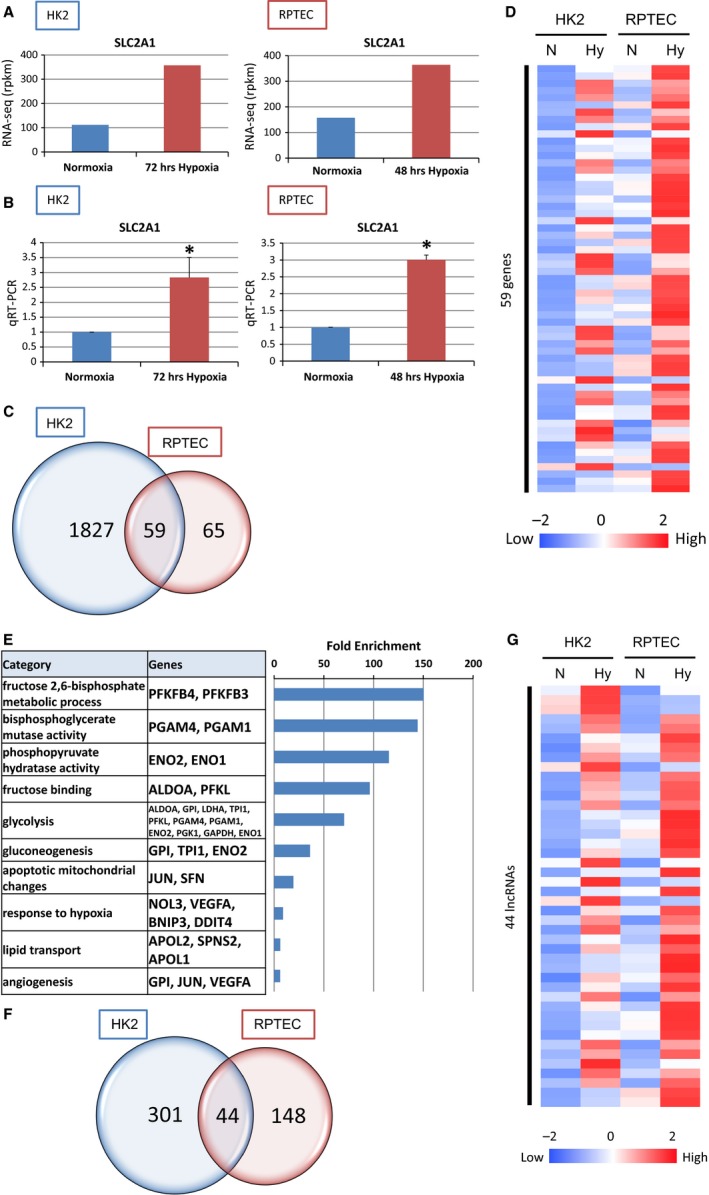
The results of RNA‐seq identified hypoxia‐inducible genes and lncRNAs under hypoxia in tubular cell lines. (A) RNA‐seq data in normoxia and hypoxia on the SLC2A1 loci in HK2 and RPTEC. HK2 were exposed to hypoxia for 72 h and RPTEC for 48 h. (B) Quantitative RT‐PCR of expression of SLC2A1 to validate RNA‐seq data in HK2 and RPTEC. (C) Venn diagram of hypoxia‐inducible genes in HK2 and RPTEC from RNA‐seq data. Thousand eight hundred and twenty‐seven genes were up‐regulated under hypoxia only in HK2, 65 genes only in RPTEC, and 59 genes in both HK2 and RPTEC. (D) HK2 and RPTEC were stimulated by hypoxia. RNA‐seq data are summarized by heatmap. Fifty‐nine genes were up‐regulated in both HK2 and RPTEC. (E) Functional annotations for commonly up‐regulated genes and the representative gene symbols for each category are shown in the middle panels. The enrichment scores of each category from DAVID are shown in the bar graphs on the right panels. (F) Venn diagram of hypoxia‐inducible lncRNAs in HK2 and RPTEC from RNA‐seq data. The numbers indicate the HK2 and RPTEC common and unique up‐regulated lncRNAs under hypoxia. (G) RNA‐seq data are summarized by heatmap. Forty‐four lncRNAs were up‐regulated in both HK2 and RPTEC.

We performed RNA‐seq using a human tubular epithelial cell line (HK2: human kidney‐2) and primary cultured tubular cells (RPTEC: renal proximal tubular epithelial cells) under normoxia and hypoxia. To validate the results, we selected well‐known downstream target genes of HIF‐1, *SLC2A1* (solute carrier family 2A1, also known as *GLUT1*; glucose transporter 1). Up‐regulation of *SLC2A1* under hypoxic condition shown by RNA‐seq in both tubular cells (Fig. 1A) was confirmed by RT‐qPCR (Fig. 1B).

In order to identify hypoxia‐inducible genes and lncRNAs in tubular epithelial cells, we listed gene set probes that exhibited significant expression (Table S1). We set the criteria for the downstream HIF‐1 targets at >1.5 log fold up‐higher under hypoxia than under normoxia. In addition, RPKM value of hypoxic condition is >20.0. We employed ‘1.5’ as the threshold because it included many already known HIF‐1 target genes. One thousand eight hundred and eighty‐six genes met the criteria in HK2 and 124 genes in RPTEC. Fifty‐nine genes are commonly up‐regulated under hypoxia in both tubular cells including *ALDOA* (aldolase A), *DDIT4* (DNA damage inducible transcript 4), *ENO1* (enolase1), *PFKFB3* (6‐phosphofructo‐2‐kinase/fructose‐2, 6‐biphosphatase 3), *SLC2A1*, and *VEGFA* (vascular endothelial growth factor A) (Fig. 1C). The hypoxia‐inducible genes only in HK2 are as follows, ACP5 (acid phosphatase 5, tartrate resistant), EFNA1 (ephrin A1), and KRT17 (keratin 17). The up‐regulated genes which are specific in RPTEC are FABP3 (fatty acid binding protein 3), GBE1 (1,4‐alpha‐glucan branching enzyme 1), and MUC1 (mucin 1, cell surface‐associated). The expression patterns of these common up‐regulated genes are described in the heat map (Fig. 1D). Each value of the heatmap is the Z‐score for each gene on the four conditions. Many of these genes are well‐known HIF‐1 downstream target genes such as *ALDOA*,* BNIP3* (BCL2/adenovirus E1B 19 kDa interacting protein 3), *ENO1, ENO2* (enolase2), *PGAM1* (phosphoglycerate mutase 1), *SLC2A1*, and *VEGFA*. To explore the biological processes related to these genes, we subjected them to ontology analysis by Database for Annotation, Visualization and Integrated Discovery (DAVID) (http://david.abcc.ncifcrf.gov/) (Fig. 1E). Fructose 2,6‐bisphosphate metabolic process, bisphosphoglycerate mutase activity, phosphopyruvate hydratase activity, fructose binding, glycolysis and response to hypoxia are enriched in the hypoxic condition, which are in consistency with typical HIF‐1 downstream target genes.

Next, we analyzed the lncRNAs which are up‐regulated under hypoxic condition. To select lncRNAs regulated by HIF‐1, we set the criterion of >1.5 log fold up‐higher under hypoxia than under normoxia as listed in Table S1. Three hundred and forty‐five lncRNAs met the criteria in HK2 and 192 lncRNAs in RPTEC (Fig. 1F). Forty‐four lncRNAs are commonly up‐regulated under hypoxia in both tubular cells including MIR210HG, DARS‐AS1 (DARS antisense RNA1), KANSL1‐AS1 (KANSL1 antisense RNA 1), and INAFM2 (InaF motif containing 2). The hypoxia‐inducible lncRNAs only in HK2 are as follows: BISPR (BST2 interferon stimulated positive regulator), DGCR5 (DiGeorge syndrome critical region gene 5), and ERICD (E2F1‐regulated inhibitor of cell death). The up‐regulated genes which are specific in RPTEC are ARAP1 (ArfGAP with RhoGAP domain, Ankyrin repeat and PH domain 1)‐AS1 (antisense 1), FOXN3 (forkhead box N3)‐AS1 and HOXC (homeobox C cluster)‐AS3 (anti‐sense RNA3). The expression patterns of these common up‐regulated lncRNAs are described in the heat map (Fig. 1G).

### DARS‐AS1 is a novel HIF‐1 downstream target lncRNA under hypoxia

**Figure 2:**
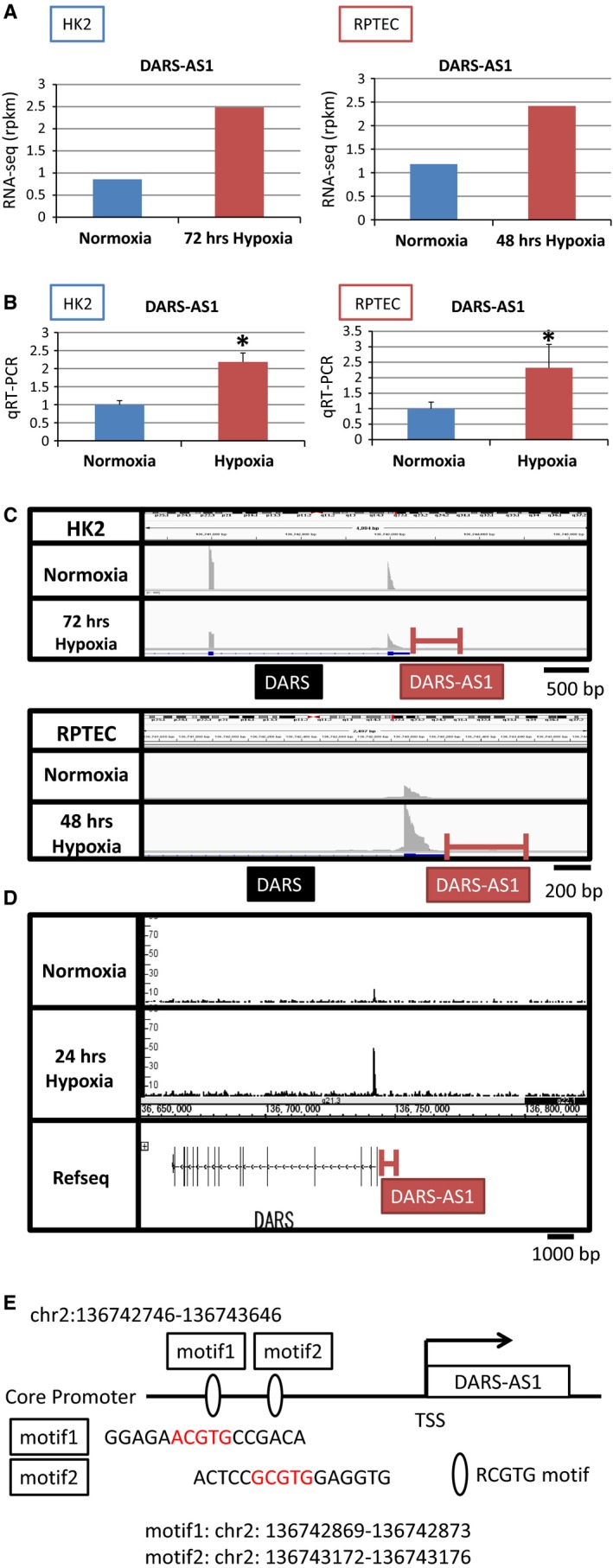
DARS‐AS1 is hypoxia‐inducible lncRNA identified using RNA‐seq and ChIP‐seq. (A) RNA‐seq data in normoxia and hypoxia on the DARS‐AS1 loci in HK2 and RPTEC. HK2 were exposed to hypoxia for 72 h and RPTEC for 48 h. (B) Quantitative RT‐PCR of expression of DARS‐AS1 to validate RNA‐seq data in HK2 and RPTEC. (C) RNA‐seq data of HK2 and RPTEC under normoxia and hypoxia on the loci of DARS and DARS‐AS1. (D) ChIP‐seq data of HK2 under normoxia and hypoxia on the loci of DARS and DARS‐AS1. (E) Schematic representation of the DARS‐AS1 promoter region and RCGTG motifs. There are two RCGTG motifs shown in red in the core promoter of DARS‐AS1.

Among commonly up‐regulated lncRNAs in Figure 1F, we validated the results of RNA‐seq by qRT‐PCR (data not shown). We focused on the role of DARS‐AS1 (aspartyl‐tRNA synthetase anti‐sense 1) because its function is almost unknown although its signal‐noise ratio is clear and the change of its expression is remarkable compared with other lncRNAs. The result of RNA‐seq showed that the RPKM of DARS‐AS1 is commonly up‐regulated under hypoxia in both tubular cells (Fig. 2A). The findings were subsequently validated by RT‐qPCR (Fig. 2B). According to the database of NCBI, the location of DARS‐AS1 (NR_110199) is positioned at chr2: 136742746‐136765112. DARS‐AS1 is anti‐sense of DARS (aspartyl‐tRNA synthetase), however, its role is not known especially under hypoxia. The results of RNA‐seq showed that the increased reads near the promoter region of *DARS* were common in DARS‐AS1 in both tubular cells (Fig. 2C). The results of ChIP‐seq of HIF‐1 using HK‐2 demonstrated that HIF‐1 binds to the promoter regions of *DARS* and *DARS‐AS1* especially under hypoxia (Fig. 2D). We examined the promoter regions of DARS‐AS1 and found two RCGTG motifs, Hypoxia‐Responsive Elements (HREs), (Fig. 2E). These results suggested that HIF‐1 binds to the HREs in the promoter region of *DARS‐AS1* and up‐regulates its expression under hypoxia.

### The expression of DARS‐AS1 is dependent on hypoxic conditions and HIF‐1 regulation

**Figure 3:**
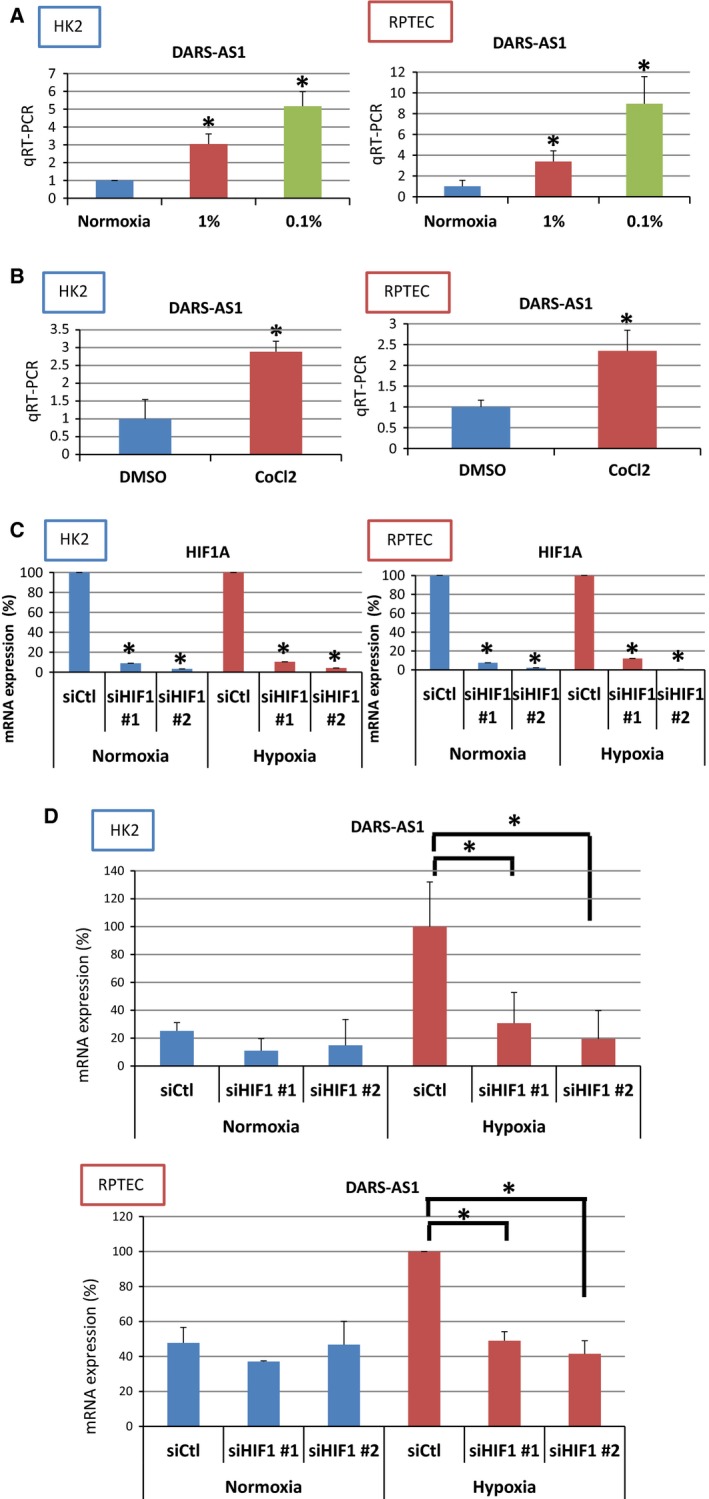
DARS‐AS1 is hypoxia‐inducible lncRNA regulated by HIF‐1*α*. (A) Quantitative RT‐PCR showed a concentration‐dependent increase in DARS‐AS1 under hypoxia both in HK2 and RPTEC. (B) Quantitative RT‐PCR showed increase in DARS‐AS1 by stimulation of cobalt chloride both in HK2 and RPTEC. (C) The mRNA level of HIF1A using two different sequences of siRNA for HIF1A in HK2 and RPTEC. (D) The mRNA level of DARS‐AS1 using two different sequences of siRNA for HIF1A in HK2 and RPTEC.

To examine the hypoxia‐inducible expression changes of DARS‐AS1, we exposed HK2 and RPTEC to 1% hypoxia and anoxia (0.1%). The results of RT‐qPCR of both cells demonstrated that the expressions of DARS‐AS1 were up‐regulated in an oxygen tension‐dependent manner (Fig. 3A). We also examined whether the up‐regulation by hypoxic condition was dependent on HIF‐1 by using hypoxia‐mimic compounds, cobalt chloride hexahydrate. Cobalt chloride is known to work as a chemical compound of hypoxia‐mimic condition and stabilize HIF‐1 even under normoxia. The expressions of DARS‐AS1 were significantly up‐regulated under the exposure of 300 *μ*mol/L of cobalt chloride for 16 h in both cells (Fig. 3B). Next, we confirmed HIF‐1‐dependent regulation of DARS‐AS1 by knockdown of HIF‐1. The knockdown efficiency of HIF‐1A is 91.1% (oligo1) and 96.8% (oligo2) in HK‐2, 89.6% (oligo1) and 95.8% (oligo2) in RPTEC (under normoxia) and 89.6% (oligo1) and 95.8% (oligo2) in HK‐2, 88.9% (oligo1) and 99.5% (oligo2) in RPTEC (under hypoxia), respectively (Fig. 3C). The expressions of DARS‐AS1 under hypoxia when HIF‐1A was knocked down were reduced by 75.5% (oligo1) and 72.4% (oligo2) in HK2 and by 51.0% and 59.5% in RPTEC, respectively (Fig. 3D). These results showed that DARS‐AS1 is up‐regulated by HIF‐1*α*.

### DARS‐AS1 inhibited apoptotic cell death under hypoxia

**Figure 4:**
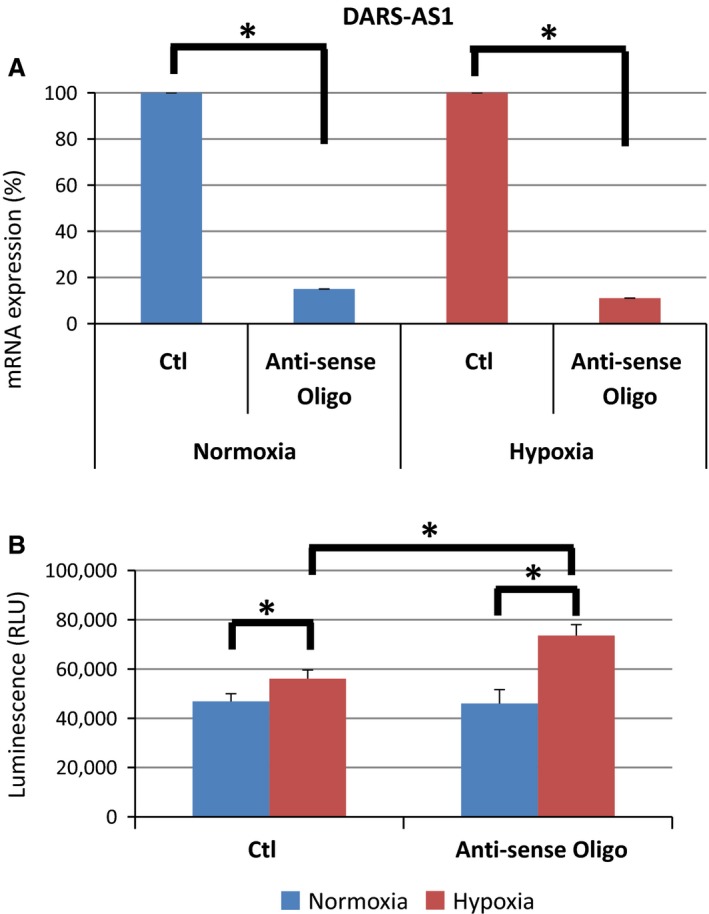
Knockdown of DARS‐AS1 aggravates apoptotic cell death. (A) The mRNA level of DARS‐AS1 using anti‐sense oligo under normoxia and hypoxia. (B) Caspase 3/7 assay under normoxia and hypoxia in HK2 using anti‐sense oligo of DARS‐AS1 showed the increased apoptotic cell death under hypoxia.

Tubular epithelial cells undergo a process of morphological changes for the ability of proliferation, deletion of polarity, and apoptotic cell death, leading to tubulointerstitial fibrosis. An increasing number of papers that report lncRNAs regulate apoptosis has been recently published (Chen et al. 2016; Luo et al. 2016; Yin et al. 2016). In this study, we examined whether DARS‐AS1, a novel lncRNA regulated by HIF‐1, affects apoptosis in tubular cells. We performed the apoptotic assay using knockdown of DARS‐AS1 by anti‐sense oligo. The level of mRNA for DARS‐AS1 is reduced 85% under normoxia and 89% under hypoxia, respectively (Fig. 4A). Under hypoxic condition, caspase3/7 activity was up‐regulated significantly, while the activity additionally increased when the DARS‐AS1 was knocked down (Fig. 4B).

We also examined caspase3/7 activity using another type of cell line, HEK293, in order to confirm whether the function of DARS‐AS1 is applicable not only in renal epithelial cells but also other cell types. We confirmed the results utilizing another type of cell line. (Fig. S1).

## Discussion

To identify novel lncRNAs which are induced by hypoxia with important biological significance, we performed RNA‐seq using two different tubular cells. There have been some reports using RNA‐seq to identify lncRNAs in the kidney. Arvaniti et al. (2016) performed RNA‐seq using unilateral ureteral obstraction (UUO) model mice. They confirmed the transcriptional activity of lncRNAs and demonstrated that three lncRNAs (*RP23‐45G16.5* transcript, *3110045C21Rik, and AI662270)* were up‐ or down‐regulated in some mouse models of nephropathies in addition to UUO. They also showed that one selected lncRNA (*3110045C21Rik)* can influence the expression of fibrosis‐related proteins in an in vitro study. Overexpression of lncRNA in the mesangial cells was reported to affect fibrosis. Wang et al. (2016) found that one lncRNA, which is associated with Cyp4a12a, was decreased in diabetic nephropathy and that its overexpression in mouse mesangial cells reversed proliferation and fibrosis of diabetic nephropathy. In our study, we used tubular cells and found that DARS‐AS1 can inhibit apoptotic cell death under hypoxia, suggesting that DARS‐AS1 might be important in the renal injury.

There is another report focusing on the role of hypoxia‐inducible lncRNAs. In ischemia‐reperfusion injury model mice, lncRNA named PRINS (Psoriasis susceptibility‐related RNA Gene Induced by Stress), was significantly up‐regulated in hypoxic condition and had specific interaction with RANTES (Regulated on activation, normal T‐cell expressed and secreted) (Yu et al. 2016). RANTES was known to promote inflammatory responses in acute kidney injury. The authors demonstrated that PRINS regulated by HIF‐1*α* might be involved in RANTES production in renal tubular cells. DARS‐AS1 has a possibility that it inhibits renal fibrosis, while PRINS had a role of promoting acute kidney injury.

Using genome‐wide analysis, we identified hypoxia‐inducible 44 lncRNAs which are commonly up‐regulated in both tubular cells. Among them, DARS‐AS1 is a lncRNA as anti‐sense of DARS and there has been no report about it as far as we searched. The functions or roles of DARS also remain largely unknown. DARS has two types, cytoplasmic DARS (also known as DARS1) and mitochondrial DARS known as DARS2. DARS2 is a mitochondrial enzyme which specifically aminoacylates aspartyl‐tRNA. Mutations of DARS2 are associated with leukoencephalopathy with brainstem and spinal cord involvement and lactate elevation (van Berge et al. 2012; Martikainen et al. 2013; Berge et al. 2014). Homozygous mutations in cytoplasmic DARS were reported to cause hypomyelination with brain stem and spinal cord involvement and leg spasticity (Taft et al. 2013). The authors suggested that mutations in cytoplasmic DARS can cause a broad range of neurologic disorders as well as DARS2. The crystal structure of cytoplasmic DARS is a homodimer and the phosphorylation of Ser146 provoked the separation of cytoplasmic DARS from the multi‐tRNA synthetase complex (Kim et al. 2013). Cytoplasmic DARS is also reported to interact with elongation factor 1 alpha (EF1A) (Reed et al. 1994; Guzzo and Yang 2008) and elongation factor 1 delta (EF1D) (Sang Lee et al. 2002).

DARS‐AS1 is located as the antisense of cytoplasmic DARS. The role of anti‐sense RNA strand is known to bind to the sense strand and inhibit translation of the sense strand. However, recent papers have shown that antisense RNAs regulate or promote the expressions of the sense transcripts (Faghihi et al. 2008; Lv et al. 2016; Zhu et al. 2016). Considering our results, there is a possibility that DARS has a role of regulating apoptosis because we found a novel role of DARS‐AS1 as an inhibitor of apoptotic cell death. Further experiments should be needed to clarify the relationship between DARS and DARS‐AS1 expression, and give us new insights about how phenotypic effects are influenced.

There are some limitations in this study. It is difficult to demonstrate the importance of DARS‐AS1 in disease model mice because we identified DARS‐AS1 as a novel lncRNA in human species. We cannot find DARS‐AS1 in mice refseq at the current moment. There is a possibility that DARS‐AS1 does not exist in mice, and we cannot prove the expression of DARS‐AS1 in the kidney of chronic kidney disease model. However, it may be more important to identify novel therapeutic targets in human species than in mice because we would like to develop novel drugs and improve our patients with chronic kidney diseases. In terms of that view, this study is of great value to find a novel lncRNA which plays important roles under hypoxia in human species.

In summary, we demonstrated that DARS‐AS1 is a novel lncRNA regulated by HIF‐1 in human renal tubular cells and found that DARS‐AS1 inhibits cell death under hypoxia. Identification of novel HIF‐1 downstream target lncRNAs helps our understanding of pathogenic mechanisms of kidney injury and may lead to discovery of new therapeutic modalities against tubulointerstitial hypoxia.

## Conflict of Interest

None declared.

## Data Accessibility

## Supporting information




**Figure S1.** Caspase 3/7 assay under normoxia and hypoxia in HEK293 using anti‐sense oligo of DARS‐AS1 demonstrated the reduction of live cells under hypoxia.

**Table S1.** Lists of hypoxia inducible genes and lncRNAs in HK2 and RPTEC.
