# Supplementary material for: Novel lnc RNA regulated by HIF‐1 inhibits apoptotic cell death in the renal tubular epithelial cells under hypoxia
Source: Physiol Rep. 2017 Apr 18;5(8):e13203. doi: 10.14814/phy2.13203 (PMC5408278; doi:10.14814/phy2.13203)
Supplement: Supplementary file 1 — Figure S1. Caspase 3/7 assay under normoxia and hypoxia in HEK293 using anti‐sense oligo of DARS‐AS1 demonstrated the reduction of live cells under hypoxia. [file PHY2-5-e13203-s001.pptx]

## Slide 1
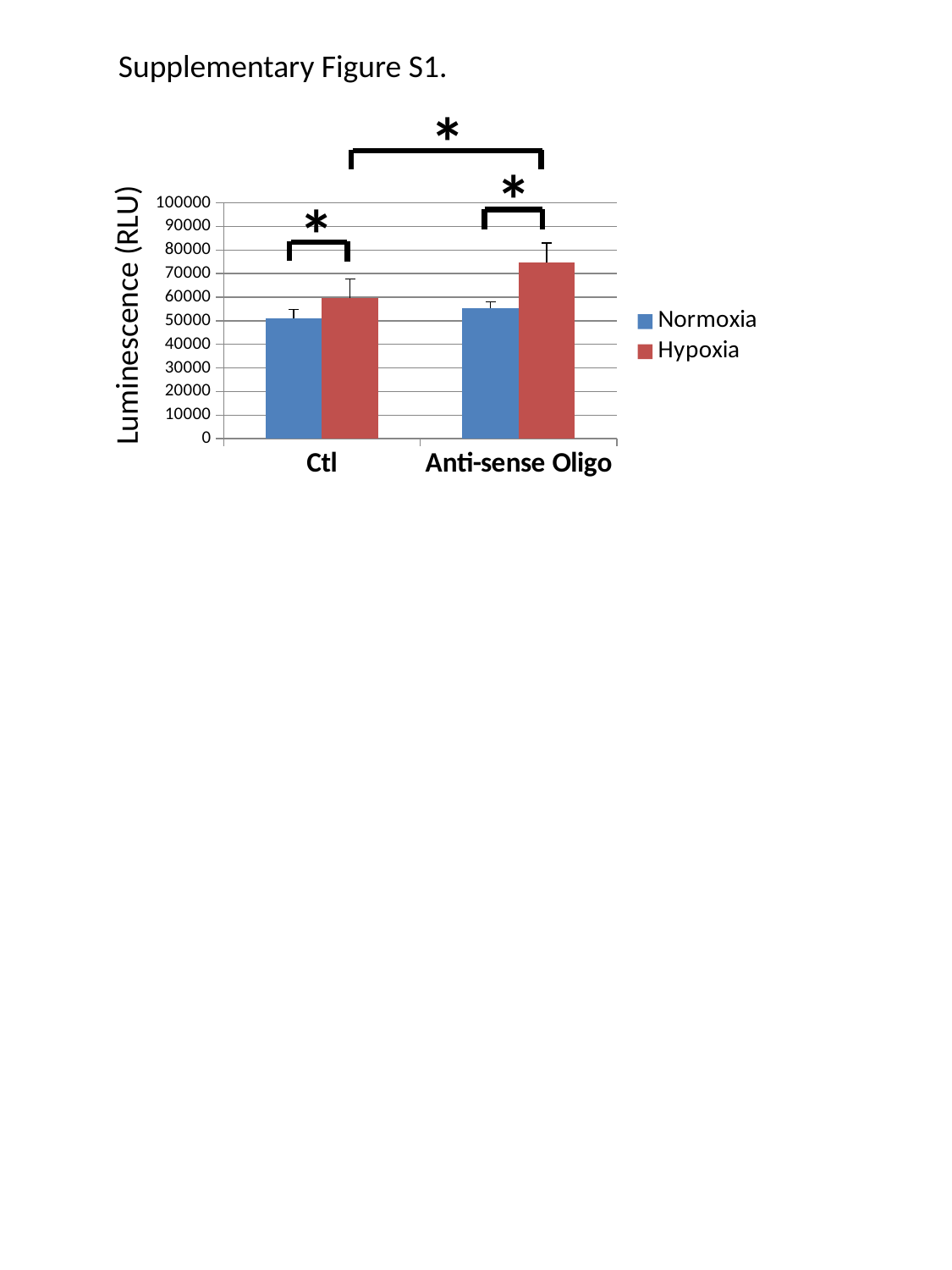

Supplementary Figure S1.
*
*
*
### Chart
| Category | Normoxia | Hypoxia |
|---|---|---|
| Ctl | 50921.666666666664 | 59510.0 |
| Anti-sense Oligo | 55508.333333333336 | 74625.0 |
Luminescence (RLU)
