# Supplementary material for: Novel lnc RNA regulated by HIF‐1 inhibits apoptotic cell death in the renal tubular epithelial cells under hypoxia
Source: Physiol Rep. 2017 Apr 18;5(8):e13203. doi: 10.14814/phy2.13203 (PMC5408278; doi:10.14814/phy2.13203)
Supplement: Supplementary file 2 — Table S1. Lists of hypoxia inducible genes and lncRNAs in HK2 and RPTEC. [file PHY2-5-e13203-s002.pptx]

## Slide 1
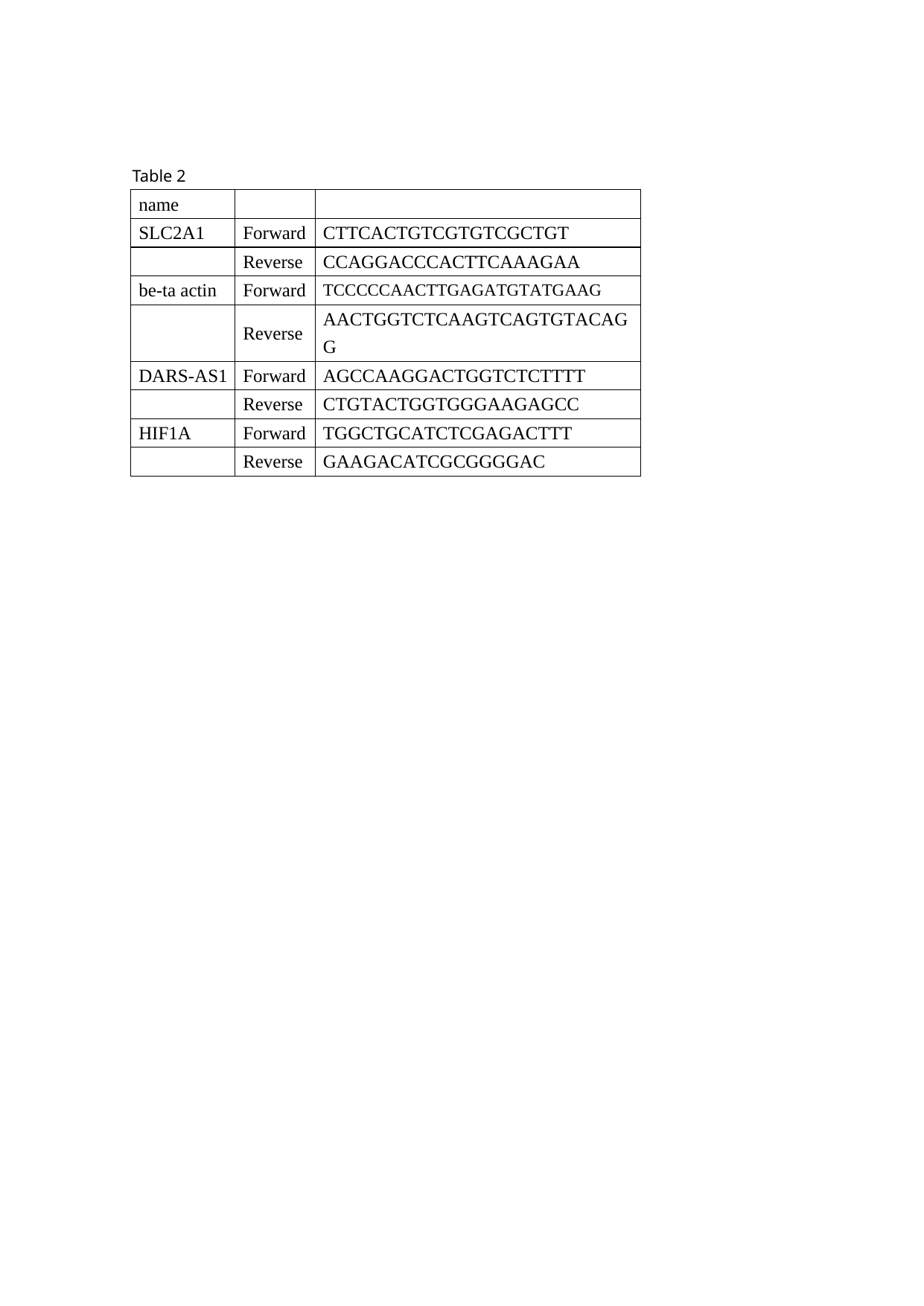

Table 2
| | | |
| --- | --- | --- |
| name | | |
| SLC2A1 | Forward | CTTCACTGTCGTGTCGCTGT |
| | Reverse | CCAGGACCCACTTCAAAGAA |
| be-ta actin | Forward | TCCCCCAACTTGAGATGTATGAAG |
| | Reverse | AACTGGTCTCAAGTCAGTGTACAGG |
| DARS-AS1 | Forward | AGCCAAGGACTGGTCTCTTTT |
| | Reverse | CTGTACTGGTGGGAAGAGCC |
| HIF1A | Forward | TGGCTGCATCTCGAGACTTT |
| | Reverse | GAAGACATCGCGGGGAC |
